# Standard Patient History Can Be Augmented Using Ethnographic Foodlife Questions

**DOI:** 10.3390/nu15194272

**Published:** 2023-10-06

**Authors:** June Jo Lee, John Wesley McWhorter, Gabrielle Bryant, Howard Zisser, David Miles Eisenberg

**Affiliations:** 1 Food Ethnographer LLC, San Francisco, CA 94109, USA; gab@foodethnographer.com; 2 Suvida Healthcare, Houston, TX 77009, USA; j.wesley.mcwhorter@gmail.com; 3 Supersapiens, Atlanta, GA 30318, USA; hzisser@gmail.com; 4 Culinary Nutrition, Department of Nutrition, Harvard T H Chan School of Public Health, Boston, MA 02115, USA; deisenbe@hsph.harvard.edu

**Keywords:** patient history, patient interviewing, food history, food diary, relationship to food, food is medicine, teaching kitchens, ethnography, food recall, diet history

## Abstract

The relationship between what and how individuals eat and their overall and long-term health is non-controversial. However, for decades, food and nutrition discussions have often been highly medicalized. Given the significant impact of poor nutrition on health, broader discussions about food should be integrated into routine patient history taking. We advocate for an expansion of the current, standard approach to patient history taking in order to include questions regarding patients’ ‘foodlife’ (total relationship to food) as a screening and baseline assessment tool for referrals. We propose that healthcare providers: (1) routinely engage with patients about their relationship to food, and (2) recognize that such dialogues extend beyond nutrition and lifestyle questions. Mirroring other recent revisions to medical history taking—such as exploring biopsychosocial risks—questions about food relationships and motivators of eating may be essential for optimal patient assessment and referrals. We draw on the novel tools of ‘foodlife’ ethnography (developed by co-author ethnographer J.J.L., and further refined in collaboration with the co-authors who contributed their clinical experiences as a former primary care physician (D.M.E.), registered dietitian (J.W.M.), and diabetologist (H.Z.)) to model a set of baseline questions for inclusion in routine clinical settings. Importantly, this broader cultural approach seeks to augment and enhance current food intake discussions used by registered dietitian nutritionists, endocrinologists, internists, and medical primary care providers for better baseline assessments and referrals. By bringing the significance of food into the domain of routine medical interviewing practices by a range of health professionals, we theorize that this approach can set a strong foundation of trust between patients and healthcare professionals, underscoring food’s vital role in patient-centered care.

## 1. Introduction

We imagine that sometime in the near future healthcare providers will ask a series of questions about their patients’ relationship to food as part of a routine history and physical examination. They will see food as a portal into their patients’ lives that will lead to better clinical assessments, informed referrals, and trust building, which all support improved health outcomes for patients, as well as higher job satisfaction for health care providers. 

We propose a minimally demanding approach to the complex task of assessing diet related health risks and ‘foodlife’ (total relationship to food) through the introduction of a novel set of questions into the standard patient history questionnaire used by health professionals. The goal of these questions is to further enhance timely referrals to resources and specialists, as well as to strengthen the connection between patient and provider through deep curiosity, non-judgmental empathy, mutual understanding, and enjoyment in the patient–provider relationship.

**Figure 1:**
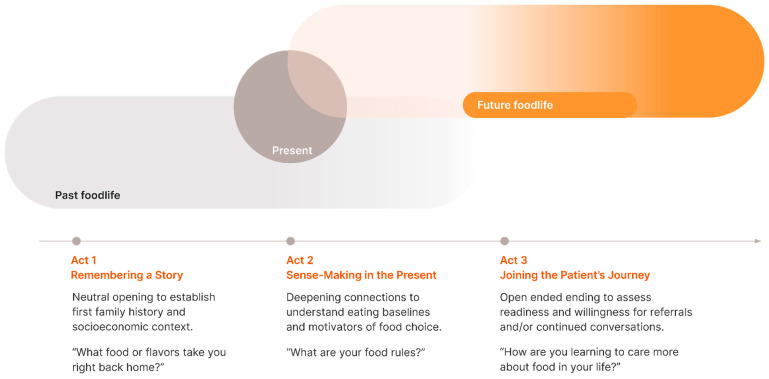
Taking a Foodlife History in Three Acts. We propose a model set of foodlife questions organized into three acts along a timeline of past, present, and future.

As in a good story or play, foodlife questions are structured in three acts, and can be completed in 5–7 min (see Figure 1). The series of open-ended questions we propose will drop the patient into storytelling along a timeline of past, present, and future:

Act 1. “What food or flavors take you right back home?”
Act 2. “What are your food rules?”
Act 3. “How are you learning to care more about food in your life?”

From here, providers can join their patients’ foodlife journeys with follow ups and increased customized referrals to registered dietitian nutritionists (RDNs), social workers, mental health therapists, teaching kitchen chefs and instructors, AI-driven cooking platforms (with customized risk and preference filters), food coach apps (such as Noom), Continuous Glucose Monitoring (CGM) devices, Cognitive Behavior Therapy (CBT), weight loss programs, and more. 

Our position is based on the assumed perspective that healthcare is interdisciplinary [1], and requires knowledge sharing and collaboration across services, specialties, and sectors (healthcare and food industry) in order to deliver the most effective patient-centered care [2,3]. To encourage patients to share a meaningful narrative about their dietary habits, we present a theoretical ethnographic foodlife set of questions adapted for healthcare providers, to augment the standard patient history relating to food.

### 1.1. Background

While calling for an expansion of the standard patient intake may strike some as unnecessarily burdensome—requiring adjustments from medical school curricula to training and protocols for frontline clinical staff—we remind readers that similar changes have been readily and effectively made in recent history. Review of recent literature regarding additions to standard intake include, but are not limited to: screening questions for smoking habits [4], use of complimentary therapies in medicine [5], experience with intimate partner violence and abuse of elderly and vulnerable adults [6], and suicide risk [7]. History of food insecurity screening is now also being implemented in many clinical settings [8]. This update in particular reflects the correlation of what we eat and do not eat with health—food insecurity screening is one example of bringing food relationships and nutritional needs to light in a clinical setting.

The above-mentioned refinements to the standard medical history have quickly become second nature for many practitioners, and have produced significant improvements in patient care. These screening tools are the first step in identifying patient needs and resourcing them with appropriate interventions—for example, screening tools have been able to reasonably identify women experiencing intimate partner violence [9], while emergency room screening and intervention for at-risk suicide patients has been shown to decrease post-emergency department suicidal behavior [10]. Related fields, including social work and psychology, also update standard screening questionnaires to reflect evidence-based insights into effective intake interviewing practices [11]. Training for critical thinking skills and an integrated view of the patient also lead to improved health outcomes [12,13,14]. 

### 1.2. Challenges

A challenge for conversations about food in a clinical context is that patients arrive with the baggage of previous judgments about their eating choices, a sense of providers’ expectations and decision-making power, and prior interactions regarding weight, BMI, and what ‘screening’ can imply (referral to resources or additional institutional monitoring, complications with insurance and coverage, and/or increased barriers to care). Furthermore, conversations about food are about much more than nutritional impacts or even biological health. For patients from marginalized communities in particular, questions about food may open up sensitive topics of food access and quality, body image and weight, racialized or other judgments, and/or susceptibility to and management of chronic illnesses associated with a complex of risk factors beyond an individual’s control.

The range of current obstacles facing physicians and other health professionals in talking to patients about food is broad. Care providers are often operating with a general lack of knowledge about nutrition, interviewing, and socio-cultural awareness of patients’ day-to-day realities [12]. A lack of consistent documentation of clinical food interviews also frustrates provider efforts to sustain the topic [15]. 

A primary obstacle, however, is that interviewing is “one of the most difficult clinical skills to master,” and its “demands made on the physician are both intellectual and emotional. The analytical skills of diagnostic reasoning must be balanced with the interpersonal skills needed to establish rapport with the patient and facilitate communication” [16]. Effective interviewing can be of “greater diagnostic value than either the physical examination or results of laboratory investigations,” [16] but requires analytic and emotional skills, balancing empathy and human concern with diagnostic pattern recognition. This skill set is also required of ethnographic researchers, and informs the interdisciplinary appeal here. The strategies summarized below are intended to augment the standard patient history relating to food.

## 2. Methods and Materials

### 2.1. Foodlife Ethnographic Interviewing 

Ethnographic interviewing (EI) encompasses a set of qualitative research methods defined by non-judgmental, integrative, and discursive documentation of the individual’s self-reported experiences [17,18]. The interviewer holds an internal state of deep curiosity and care in order to actively listen for insights. They trust people to be expert guides of their own lives and/or constructed realities (worlds). 

Foodlife describes the broad and complex relationships to food, and how eating patterns change over time. ‘Foodlife’ is a concept coined by co-author June Jo Lee to understand motivators of food choice and how to influence food consumption, from almost two decades of proprietary ethnographic field research about health and wellness for the food industry (packaged foods, food retail, and foodservice) with US adults representing the general population. Foodlife EI is a portal to quickly understand people’s past–present–future orientations: troubles (anxieties; ambiguities), appetites (needs; desires), and aspirations (dreams; trajectories). Edibility, or what’s ‘good to eat,’ relates to both biological and cultural filters for what individuals and society deem as good, tasty, and healthy. For example, whether or not edible insects are good-to-eat depends on one’s cultural identity and/or orientation to climate change. In so many ways, we are what we eat and do not eat. As French gastronome Jean Anthelme Brillat-Savarin wrote—about what would now be understood as biopsychosocial health and character determined by what one eats—“Tell me what you eat, and I will tell you what you are.” [19]. 

While we are not recommending that all physicians and licensed health professionals be trained as ethnographers (at least, not outright), we offer concise ethnographic foodlife questions and techniques to support effective clinical interviewing. 

### 2.2. Foodlife in Three Acts

Like other updates to the standard patient intake (‘history’) that screens for biopsychosocial risks, we propose that Foodlife EI—as adapted here for clinical encounters—is a critical update to improve patient assessment and care. Foodlife questions are structured along a timeline of the patient’s past, present, and future. As the provider asks questions to move the patient through the timeline, the goal is to build rapport and learn from the patient; while in the background, the provider makes sense of the patient’s motivators, eating patterns, and where they may be open—or closed—to explore a new relationship to food. Narrative Medicine offers a parallel concept of using storytelling and empathy “to rehumanize medicine.” [20]. 

We present several ways to ask the proposed questions (see Table 1). The provider can choose a way that feels most comfortable for the patient. If the patient seems stuck and unable to answer, the provider can try out other questions in rapid succession, thereby enabling the patient to choose how they want to answer. Providers can also model prospective answers (“for example, my mom’s kimchi takes me right back home”). In some instances, providers may also observe that this line of inquiry will not be productive at this moment in time.

#### 2.2.1. Prologue. “Can I Ask You a Few Questions about Your Relationship to Food?”

Foodlife EI begins with the patient’s consent to talk about their relationship to food. Temporarily suspend, as much as possible, your own judgments, biases, and agenda. Trust the patient as expert guides of their own worlds. Actively listen with eye contact, note-taking, and short follow up prompts for clarifications. 

The essential value of active listening with eye contact and note taking is to build trust within the brief clinic visit. They effectively signal, ‘I’m paying attention to you, and what you’re saying is important’. Current medical trainees frequently fall into the habit of not looking at the eyes of their patients. To the patient, younger clinicians who do not maintain eye contact may appear as extensions of computers, filling in electronic medical records. To ask exploratory and deeply personal questions about food without eye contact returns a diminished value to sharing, and a diminished relationship with the patient. 

Proceed with consent, curiosity, and genuine eye contact.

#### 2.2.2. Act 1. “What Food or Flavors Take You Right Back Home?”

Alternative ways to invite the patient to share a story about food:

- “What were your familiar tastes or foods growing up?”
- “Using all your senses, remember an unforgettable bite of food. Can you describe it?”
- “Can you describe the first time you made something delicious just for you?”

Sample follow up prompts focus on positive sensations, feelings, and details:

- “What did it taste/smell/feel like?”
- “Who else was there? Who prepared the food?”
- “What was the mood around the table as you ate?”

Now the provider knows something from the patient’s early memories and relationship to food in the context of family and culture. Storytelling drops the patient and provider into an emotional register of relating person–person rather than patient–provider; neutralizes potential shame by centering on the patient’s own experience rather than impersonal ‘clinical’ metrics (BMI, lipid panel, and A1C test results); and gives the patient control over continuing or deferring the conversation. 

The opportunity for the provider is to deeply feel (tune into and mirror) the patient’s world (constructed reality). Anchoring in the patient’s past, this step offers many narrative threads to learn more about first family’s orientation to food, cultural foods, and early-life social determinants of health (why someone is eating something or not, and what the best options are moving forward). This patient-centered line of inquiry is conducted with the aspiration of learning how best to help the patient in terms of future questions/steps/referrals that may enhance the patient’s short- and long-term physical and mental health. The ideal takeaway message: ‘You get to decide what to share, and I’m here to listen and support you in feeling better.’

Red flags: Since we are listening for what is said and not said, when the patient cannot answer the first question, they have just told you that the issue is not about food. Based on two decades of extensive foodlife interviews with consumers about health and wellness for the food industry, we have learned that it is about trauma, and/or other social determinants of health that are expressed through food. Proceed with great care by asking for a more recent happy food memory. Or, apologize and discontinue questioning. Follow up with referrals for nutritional, social, or mental health specialists as necessary and welcomed by the patient; or, simply note this and decide if this is an area worthy of revisiting at a later time and circumstance. While these sensibilities are known to experienced MD’s, RDN’s and other health professionals, it is worth highlighting them in the context of enhanced, routine patient history taking in relation to food.

#### 2.2.3. Act 2. “What Are Your Food Rules?”

Alternative ways to map out a patient’s motivators of eating:

- “What is your approach to food? Or, do you have a food philosophy?”
- “What does eating well look like? What takes you off track?”
- “Are there foods you’re seeking more of? Any foods you’re avoiding?”

Sample follow up prompts focus on the ‘how’ behind the eating:

- “How does eating this way make you feel? What does it help you do?”
- “How is this different from the way you ate growing up?”
- “How did you learn about this way of eating?”

Now, the provider senses the patient’s current beliefs and behaviors around eating. Sharing present day foodlife strategies invites the patient to trust that their provider will not suddenly shift to a blame-shame register; builds a safe space for exploration of the patient’s ‘edibility filters’ (motivators of eating—see Section 2.3); and gives the patient permission to share their actual eating patterns rather than a generic ‘trying to be healthy’ story that may obfuscate what is really going on. 

The opportunity for the provider is to observe the whole picture of the patient’s motivations behind their eating patterns, and map out their eater profile (cultural edibility filters), rather than pivot to generic ‘fixes.’ By anchoring in the patient’s daily eating habits, this step offers more information for comprehensive assessment and prioritization of needs and next steps, including the prospect and timing of suitable referrals to nutrition and diet-related specialists. The ideal takeaway message: ‘This is a continuing conversation, not a jump to judgment and intervention.’

Red flags: When the patient seems evasive or vague about how and what they eat, this may indicate disordered eating or deeper issues that are not about food per se. Proceed with great care by asking follow up prompts for details of ‘how,’ rather than asking ‘why.’ Follow up with referrals to social or mental health specialists, while also considering referrals to nutrition experts, as necessary.

#### 2.2.4. Act 3. “How Are You Learning to Care More about Food in Your Life?” 

Alternative ways to meet the patient where they are ready and willing to explore a different relationship to food:

- “Where do you want to take your relationship to food a year from now?”
- “Anything new in food you’re thinking of including in your life?”
- “Do you have a question, or worry, about food that you’ve wanted to ask a doctor or nutrition specialist but never could?”

Sample follow up prompts focus on readiness and willingness to change:

- “When do you enjoy trying something new?”
- “When do you want to start?”
- “When are you blocked?”

Finally, the provider can join the patient to take steps toward better eating. Imagining a future foodlife invites the patient to explore a different relationship to food with non-judgemental empathy; expands the patient’s own foodlife narrative beyond nutritionism or blame-shame with the provider as witness; and leads to an open-ended process, potential, and possibility for change, rather than end goals (BMI, ideal weight, better blood sugar control, etc.). 

The opportunity for the provider is to meet the patient when and where they are ready and willing to begin moving their foodlife journey toward better health outcomes and more personal agency. Anchoring in a future orientation moves the provider’s task to advocate for the most appropriate next steps; and offers the provider (as de-facto first line of inquiry) a sense of when this may be appropriate for this particular patient; and, whether and when suitable referrals can and should be suggested. The ideal takeaway message: ‘Patient care includes professional referrals as well as guidance to food resources (access, coaching, and teaching) based on when and where the patient wants to take their foodlife journey.’ 

More often than not, the patient is benefitted by taking this journey with a trusted health professional’s personal involvement and guidance. To the patient who is ready and willing to start, the provider can now say, “And so we begin this journey together.” Again, this is an approach known to most skilled clinical caregivers, but it can also be considered for more routine use in a standard history relating to food.

Red flags: As the provider continues to listen for what is said and not said, the patient may indicate lack of readiness or unwillingness to explore a different relationship to food. In this instance, the most appropriate thing to do is be patient. Accept that any recommendations relating to food may not be accepted by the patient at this time. However, this line of inquiry, if not acted on, may enhance the trust between the patient and provider. Timing is everything, and this may be a time to wait to return to this line of inquiry again at a future date, if and when, in the provider’s judgment, that seems to be in the patient’s best interest. As a reminder of a parallel to other behavioral health challenges, smoking cessation, and/or assistance with anxiety or depression, often require multiple coordinated efforts, often over a lengthy time period, to achieve sustained improvement in health and wellbeing [4,21]. However, a trusting patient–provider relationship, along with an enhanced self-reflective posture on the part of the patient, are often at the core of such successful behavior changes over time.

True behavior change begins when the patient themselves starts to see and make sense of previously unarticulated and often unconscious beliefs and patterns in their relationship to food.

### 2.3. Mapping Eater Profiles

Foodlife stories describe the patient’s eating patterns and cultural edibility filters (‘what’s good to eat and not eat’) that develop over time, within the context of shared food culture, and individual taste memory and meaning-making attached to food and eating. This is the elephant in the physical exam room, and why a pizza is not just carbs, fat, and calories, nor glorious dough, red sauce, and mozzarella cheese. Pizza can be a guilty pleasure, a marker of identity, or what is affordable and accessible. Pizza can also mark the moment you found out that your parents were getting divorced. Pizza is as much desires, anxieties, dreams, as it is nutrients. 

**Figure 2:**
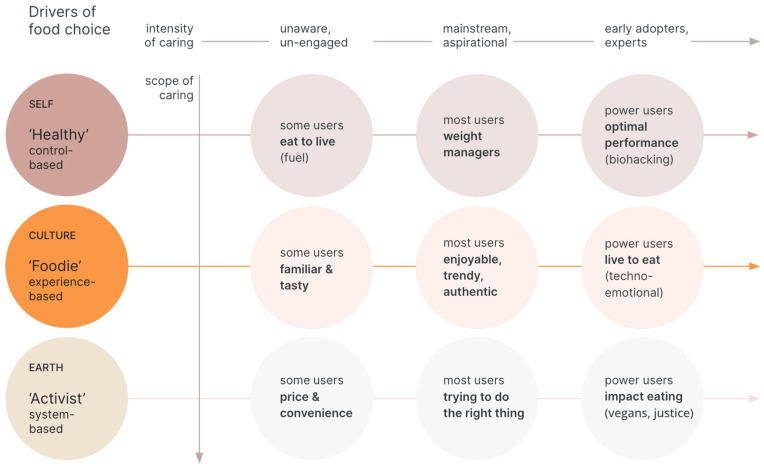
‘Good to Eat’ Cultural Framework. Developed from analysis of two decades of proprietary foodlife ethnographic interviews for the food industry. Edibility is a cultural filter. Ethnography reveals patterns of eating which may also be useful for clinicians. Using this construct, we can predict that eaters in a modern food system are distributed across three eater profiles: healthy, foodie, and activist. These ‘good to eat’ edibility filters are arranged along a *y*-axis of scope of caring (about self, community, and earth) and *x*-axis of intensity of caring (from low to high awareness and action).

Most patients are operating from their particular foodlife story within a cultural framework. The ‘Good to Eat’ Cultural Framework (Figure 2) is an analytic tool developed by co-author J.J.L. during the course of proprietary ethnographic research about health and wellness, sustainability, and premium foods for the food industry as Food Ethnographer LLC; and is inspired by an earlier iteration of The Hartman Group’s Health and Wellness World Model™ (Bellevue, WA, USA) [22] developed by a team of demographers, sociologists, anthropologists, and ethnographers (including J.J.L.) used to understand motivators of food choice for consumers’ packaged goods companies) of eating, and not from a care provider’s biomedical health risks framework. It is time to upgrade the provider’s operating system to include both, and observe where they can find the overlaps (see Figure 2). 

The provider can find the patient’s motivating ‘why’ they eat, and place them within the ‘Good to Eat’ Cultural Framework (control-based, experience-based, and system-based motivators of food choice). This provides the provider leverage to strategize possible entry points for the RDN, mental health specialist, or social worker to help the patient. Is it pleasure? Do they want to save the planet? Are they managing the chaos of their lives by trying to be really healthy, whatever that means? Or, is eating a mix of pleasure, virtue, and control?

Most eaters, living in our modern industrialized food system, are weight managers with varying rates of success (some have not started yet, and others have internalized it). Eaters also eat for pleasure, connection, and self-expression. Chefs tend to fall across all three eater profiles because they are so deep into food (farm-to-table to techno-emotional cuisine while intermittent fasting in order to eat more). For gourmands, food is an experiential and intellectual pursuit of pleasure, conviviality, and study. Vegans tend to be systems-based activists who are changing the way they eat in order to change the world. Climate activists use food as a tool to change the system, perhaps to make new worlds where humans do less harm. Biohackers are at the most intense end of control-based eating and may be drinking fatty coffee, wearing cold vests, and intermittently fasting to optimize performance, or even reverse aging.

Considering these motivators of food choice may better enable health professionals to gain a deeper understanding of why and how their patients are navigating their foodlife journeys at a particular moment in time.

### 2.4. A Summary of Underlying Strategy behind Foodlife EI

Foodlife EI moves the patient–provider through time in three acts. Acts 1 and 2 are included mainly to get the patient and provider to meet up in Act 3, where transformation may begin. The provider needs to know when and where to meet the patient on their foodlife journey, or else they will never meet. The ‘when’ is where the action starts, where change is possible. This is a summary of the underlying strategy of Foodlife EI:

Act 1. Remembering a Story (“What food or flavors take you right back home?”)

The first prompt is a neutral opening to elicit a story that drops both patient and provider down into their sensing and feeling bodies as they remember/listen to a story. They can set aside the patient’s gown and the professional’s white coat to build rapport and trust. In the comfort of their own story, the patient relays extra context, which the provider picks up through active listening to what is said and not said. The first prompt drops the patient–provider into their shared humanity. The prompt is the set up for nonjudgmental empathy and mutual respect and understanding—the effects of which have been shown to improve patient satisfaction and compliance [23].

Act 2. Sense-Making in the Present (“What are your food rules?”)

The second prompt opens a portal into the patient’s foodlife. The provider enters and follows the patient, who is the expert guide of their own beliefs and behaviors around food. The provider asks for clarification without introducing bias, maps out how the patient is making sense of what’s ‘good to eat,’ and begins to picture the patient in the ‘Good to Eat’ Cultural Framework.

Patients come into the clinic with their own ideas of how to control their eating (low carb, macro balancing, gluten-free, and keto), with their bucket lists of new foods and/or dietary lifestyles to try. Many will not be able to clearly articulate their motivators. In related foodlife research, we have observed that the standard response, ‘I try to eat healthy,’ tends to gloss over or deflect conversations about eating. We encourage providers to not introduce the word ‘healthy’ into the foodlife question set, and follow up with patients who use it by asking, “what does healthy eating look like?” In the background, the provider’s task is to place the patient within the ‘Good to Eat’ Cultural Framework. The provider can wonder, ‘Is food about family, adventure, pleasure, guilt, control, identity, performance, politics? What are the motivators most likely to engage the patient?’ The provider will note the patient’s eater profile to their assessment, which will inform the appropriate pathway of referrals. 

Act 3. Joining the Patient’s Journey (“How are you learning to care more about food in your life?”)

The final prompt leads to the patient’s future foodlife journey where they can reflect on their relationship to food—where it is, has not been, and where they want it to be. It is an open-ended beginning for the provider to finally get to, ‘So what do you want to do now? Where do you want to go? When do you want to start? How can I help you?’ Foodlife EI gets the patient to that ‘when’ they want a different future state. Thus, the provider can join the patient right when and where they are ready and willing to make steps toward eating for improved health outcomes.

## 3. Anticipated Relevance and Impact 

Unlike Motivational Interviewing (MI), EI does not try to actively guide or coach to change behaviors through awareness and reminders of one’s personal motivations. EI is not a therapeutic tool. In the clinical encounter, Foodlife EI is an assessment tool to see where patients fit into cultural frameworks around edibility (healthy, tasty, virtuous), and to determine where they are most ready and willing to make changes. EI encourages open dialogue and improves vulnerability in order for providers to dispense more successful referrals to care. As such, EI may augment the healthcare provider’s ability to better understand, refer, and gain the trust of his/her patient with respect to food choices and dietary habits.

In essence, EI is a potentially useful prequel to referrals and active therapeutic interventions which are commonplace in current practice. As such, EI may augment the healthcare provider’s ability to better understand, refer, and gain the trust of his/her patient with respect to food choices and dietary habits.

Foodlife EI is not a food recall. Foodlife is about the pleasures, problems, and fantasies of food in the context of life, and may serve as a prerequisite in the development of patient–provider trust, in the realm of a patient’s relationship to food that starts with the primary care provider and continues through follow up visits and referrals to medical specialists, RDNs, mental health therapists, and other resources. Trust is built by the provider first having confidence in and trying to understand their patient’s particular perspectives. Ultimately, Foodlife EI transforms the provider from external expert or coach to trusted partner—and de facto first line of inquiry—with respect to a patient’s own self-determined foodlife journey through assessments and referrals.

## 4. Discussion

We hope health professionals consider and explore the multidisciplinary and theoretical approach outlined herein, as epidemiological evidence makes clear that many of us are not eating optimally, which subsequently affects our health [24,25,26,27,28,29] and the national health expenditure, cited at $4.3 trillion in 2021 and growing [30]. It remains a standard clinical practice, however, for conversations with patients about food to be sporadic, specialized, medicalized, improvised, and often culturally biased. Food discussions have been narrowly focused on nutrition [31,32] and metrics (weight, BMI, nutritional markers) that are not necessarily amenable to an integrated sense of the patient’s overall relationships to and identities relating to food [33,34]. 

This standard can easily reinforce the concept—for physicians, nutrition specialists, other health professionals, and patients alike—that food is a separate (or subordinate) question from other acute or chronic issues that bring a patient to see a clinician. When not presented in a conversation that recognizes the patient’s full biopsychosocial integrity, socioeconomic context, and cultural edibility filters, screening questions regarding food choices can easily amplify shame and stigma that patients, especially those from marginalized communities, are often bringing with them into the clinical encounter [35]. 

By using the Foodlife EI construct described here, perhaps conversations around food can be better centered around the patient experience, and encourage the patient to seek additional guidance. Responses by the patient may alert the provider to possible and appropriate referral pathways for the patient. Given time pressures during routine clinic visits, clinicians may have to schedule a separate follow up with patients who are at risk or have a chronic disease related to diet and lifestyle, and are ready to explore changes in self-care strategies. Red flags that surface during the Foodlife EI may alert healthcare providers to schedule a follow up and/or referral to a range of relevant specialists.

Physicians and all health professionals directly involved with patients interviewing are now invited to experiment with the first prompt, “What food or dish takes you right back home?” The question takes two minutes. Ask friends and colleagues, and then try it out with select patients who seem open to exploring their relationship to food. Enjoy how this will open up a new relationship with your patient.

Once informally adopted, there remains the need to use, refine, and formally evaluate this suggested, novel approach to taking a foodlife history. Researchers are invited to consider observational and controlled experiments to assess the utility and potential impact of this enhanced history taking approach on health outcomes. Does this approach help gather more information, build more trust, and/or lead to more referrals and enhanced clinical outcomes for patients ready for assistance in their food journey? 

## 5. Conclusions

As summarized above, this proposed line of questioning via Foodlife EI may create a positive discussion around food and nutrition, enhance a clinician’s ability to pick up cues on who needs a referral to whom, and when this can happen with a relatively high probability of patient engagement. A prescription or referral can be personalized for the patient’s specific responses. The provider can also then recommend a specialist who has shared interests or interventions aligned with the individual patient’s sensibilities and motivations.

The value proposition we hypothesize exists, and which will only be confirmed or refuted once this approach is experimented with, is that providers who use these novel foodlife questions may be more successful in helping the patient who is ready to begin in a trusting relationship with their physician, RDN, and all the other specialists to set a new course for their foodlife journey. This, in turn, may lead to better health outcomes and to significantly better communication between patients and caregivers with regard to food. 

What we are proposing is intended to augment and enhance the current history taking of nutrition specialists, endocrinologists and primary care professionals; the reader is invited to make use of the foodlife questions, and strategies which we hypothesize will enhance patient provider communications and may lead to enhanced patient behaviors and outcomes. Certain aspects of what we are proposing here already exist within expert patient interviewing but can potentially be enhanced with this new foodlife question set. While this approach is novel, it may prove to be useful and impactful, and warrants formal study and evaluation. 

## Figures and Tables

**Table 1:** Foodlife Question Set. This question set was developed by the authors as a theoretical method for applying foodlife ethnography used in consumer food research about health and wellness strategies to taking a patient’s history relating to food during routine clinical encounters.

Foodlife Question Set			Behind the Questions			
Question	Alternative Question	Follow Up Prompts	Takeaway for Patients	Opportunity for Providers	Value for Patient Care	Red Flags
Act 1. Invite the patient to share a story about food. “What foods or flavors take you right back home?”	“What were your familiar tastes or foods growing up?” “Using all your senses, remember an unforgettable bite of food. Can you describe it?” “Can you describe the first time you made something delicious just for you?”	Focus on positive sensations, feelings, and details: “What did it taste/smell/feel like? Who else was there? Who prepared the food? What was the mood around the table as you ate?”	You get to decide what to share, and I am here to listen and support you in feeling better.	To deeply feel the patient’s world. Offers many narrative threads to learn more about first family’s orientation to food, cultural foods, and early-life socio-economic status.	To learn how best to help in terms of future questions, steps, and referrals that may enhance short- and long-term physical and mental health.	When the patient cannot even answer the first question, they have just told you the issue is not about food. It is about trauma, and/or other social determinants of health that are expressed through food. Proceed with great care.
Act 2. Map out the patient’s motivators of eating. “What are your food rules?”	“What is your approach to food? Or, do you have a food philosophy?” “What does eating well look like?” “What takes you off track?” “Are there foods you’re seeking more of? Any foods you’re avoiding?”	Focus on the ‘why’ behind the eating: “How does eating this way make you feel? What does it help you do? How is this different from the way you ate growing up? How did you learn about this way of eating?”	This is a continuing conversation, not a jump to judgment and/or intervention.	To see the whole picture of the patient’s motivations behind their eating patterns, and map out their eater profile, in order to assess, prioritize and determine next steps, including suitable referrals.	To understand the patient’s current beliefs and behaviors around food, and their actual eating patterns rather than a generic “trying to be healthy” story that may obfuscate what is really going on.	When the patient seems evasive or vague, this may indicate disordered eating or deeper issues that are not about food per se. Follow up with possible referrals to social or mental health specialists, as well as with nutrition specialists.
Act 3. Meet the patient where they are ready and willing to explore a different relationship to food. “How are you learning to care more about food in your life?”	“Where do you want to take your relationship with food a year from now?” “Anything new in food you’re thinking of including in your life?” “Do you have questions about food that you’ve wanted to ask a doctor or nutrition specialist but never could?”	Focus on readiness and willingness to change: “When do you enjoy trying something new? When do you want to start? When are you blocked?”	Patient care includes professional referrals as well as guidance to food resources (access, coaching, teaching) based on when and where you want to take your foodlife journey.	To meet the patient when and where they are ready and willing to begin moving their foodlife journey toward better health outcomes and more personal agency. Offers a sense of when this may be appropriate; and, whether and when suitable referrals can and should be suggested.	To join the patient to take steps toward better eating in a way that expands the patient’s own foodlife narrative beyond nutritionism or shame-blame, and opens a possibility for change with the provider as witness and advocate.	When the patient lacks readiness or willingness to explore a different relationship to food, the most appropriate thing to do is be patient. Accept that any recommendations relating to food may not be accepted by the patient at this time.

## Data Availability

Not applicable.
